# Editorial: Pathogenesis, immune escape, prognosis, and novel management of lymphoid proliferative disorders

**DOI:** 10.3389/fimmu.2022.1063418

**Published:** 2022-11-21

**Authors:** Wei Sang

**Affiliations:** Department of Hematology, The Affiliated Hospital of Xuzhou Medical University, Xuzhou, Jiangsu, China

**Keywords:** immune escape, prognosis, lymphoid proliferative disorders, pathogenesis, lymphoproliferative disease

This Research Topic examines lymphoproliferative diseases (LPD), focusing on the pathological microenvironment, immune escape, prognosis, and the processes involved with seeking novel treatment methods. Including four editors who worked with invited reviewers, for this collection we invited potential contributors and accepted online contributions. Based on the comprehensive evaluation of the theme fit and the quality of those manuscripts, 17 contributions were accepted. These manuscripts include different types of LPD, such as hemophagocytic lymphohistiocytosis (HLH), follicular cell lymphoma (FL), peripheral T cell lymphoma (PTCL), diffuse large B cell lymphoma (DLBCL), Castleman disease (CD), extranodal natural killer/T cell lymphoma (NK/TCL), Hodgkin lymphoma (HL), and other subtypes of non-Hodgkin lymphoma (NHL).

LPDs are often driven by different causes and have great differences in pathogenesis, pathology, and genetic characteristics. Clarifying these potential cloning and transformation mechanisms and regulatory mechanisms will help us better understand these kinds of diseases. The prognosis of DLBCL in the elderly is often poor. Zhu et al. found that the elderly DLBCL is accompanied by undesirable clinical and molecular features, represented by the accumulation of oncogenic mutations and with the MYD88-like genetic subtype and immunosuppressive tumor microenvironment alterations. It is proposed that, in the future, elderly DLBCL patients need to explore new treatment methods based on these characteristics. Tumor protein 53 (*TP53*) mutation predicts an unfavorable prognosis in DLBCL. Zhang et al. demonstrated that aberrantly activated APOBEC3B can induce TP53 G/C-to-A/T mutations in DLBCL, which may lead to proliferation and drug resistance and may contribute to R/R DLBCL, providing insight into the mechanism underlying TP53 mutation in DLBCL as well as a potential target for overcoming drug resistance in this disease. Xianhuo Wang et al. explored the role of m6A regulator expression in FL. Through the analysis of the database and the verification in the clinical cohort, it was confirmed that the patients with a low level of m6A scores had poor survival, accompanied by specific gene characteristics and a high expression of PDL1, which also had certain significance for guiding the treatment of PD1 inhibitor. CD is a rare LPD. Qian et al. demonstrated that thrombocytopenia and hypoalbuminemia are independent poor diagnostic factors for CD. In addition, they also confirmed that mTOR activation was higher in CD compared to reactive lymphoid hyperplasia. It is helpful to better understand CD and explain the molecular basis of targeted mTOR therapy.

Many studies focused on the prognostic characteristics of LPD. The prognosis of CD56-negative extranodal NKT cell lymphoma is poor, especially suitable for early-stage NK/TCL. In a large retrospective study, Yang et al. proved that combined chemotherapy based on asparaginase can overcome its disadvantage, but it needs to be confirmed in future prospective clinical studies. HLH is a clinical syndrome with the clinical manifestation of immune overreaction caused by many factors. The clinical manifestations are heterogeneous. At present, there is a lack of a standardized prognosis evaluation system to guide clinical individualized treatment. Shen et al. established a novel prognosis score system (HHLWG-NPI) through multi-center data in China, which effectively distinguishes the prognosis of patients. It is valuable to guide the accurate stratification and individual treatment in adult HLH. The limitation of the study is that more complex variables such as gene mutation, sCD25, and pro-inflammatory markers were not included. Further prospective multicenter studies are urgently needed to validate the model. In another study on Epstein–Barr virus (EBV)-related HLH, Zhao Cui et al. explored the risk factors leading to the failure of induction therapy, and based on these variables, established a nomogram prognosis model. The model can better distinguish the risk and help to guide the individualized treatment of patients. In this study, VP16 is recommended as the treatment choice for high-risk patients. EBV infection is one of the main causes of HLH. EBV-associated lymphomas are more likely to be accompanied by HLH. Zhao et al. retrospectively analyzed the clinical characteristics and prognostic factors of 51 patients in a single center. They found that patients with lymphoma accompanied by HLH could obtain a better objective response rate and overall survival (OS) by anti-lymphoma treatment as soon as possible after anti-HLH was controlled. In addition, they also proved that the increase of alanine aminotransferase is a bad prognostic factor for EBV-related lymphoma with HLH. High-grade LBCL (HGBL) is a highly invasive NHL. Kong et al. explored some clinical features of HGBL through machine learning. They found that cardiovascular appearance, Ann Arbor stage, lactate dehydrogenase (LDH), and International Progressive Index (IPI) were independent risk factors for HGBL patients. In addition, in the high-IPI-risk group, CD10 expression, extranodal involvement, a high level of LDH, a high level of white blood cell (WBC), bone marrow involvement, old age, advanced Ann Arbor stage, and high SUVmax had a higher risk of death within 1 year. This research is helpful for a more accurate and high-level individualized evaluation and treatment selection.

In terms of innovative treatment, several studies were focused on chimeric antigen receptor T cells (CAR-T) treatment. In the PD1 inhibitor and BV era, there is still an unmet need for the treatment of r/r cHL. Sang et al. explored the feasibility of targeting CD30 CAR-T cell treatment and the optimized combination therapy strategy in r/r cHL. They proved that the PD1 inhibitor combination could enhance the efficacy of anti-CD30 CAR-T cell therapy. In this study, a 50% CR rate was obtained in general, while the combination of PD1 inhibitors can reach an 80% CR rate, and the 5-year OS reached 70%. It provides a new choice for the treatment of r/r cHL. CAR-T cell treatment is important for r/r B-ALL, but there are still treatment failures and relapses after CAR-T cell treatment. In a prospective study, Gu et al. developed and validated the predictive models for CAR-T clinical responses in r/r B-ALL patients and further confirmed the diagnostic value of the risk model, which helps to screen more suitable patients and provide some reference for solving the problem of CART cell therapy failure in the future. Wang et al. explored the causes of the primary failure of CAR-T cell treatment. Through clinical variable analysis, they proved that a higher LDH level and a lower cytokine release syndrome (CRS) were independent prognostic factors for T cell dysfunction. In addition, they also explored the genetic characteristics of patients with treatment failure through full exon sequencing. This study is helpful to explain the potential mechanism of primary failure of CAR-T cell therapy and has certain significance for solving the problem of failure of CAR-T cell therapy in the future. CRS and ICANS are the main complications of CAR-T cell therapy. At present, the use of tocilizumab mainly refers to the CRS level, but some patients still progress to severe CRS. The research of Zhang et al. proved that patients with less than four increases in IL-6 levels had a higher incidence of severe CRS after receiving tocilizumab (37.5% *versus* 0%, *p* = 0.0125), which provided a basis for referring the CRS intervention strategies under the guidance of IL-6 level. It is helpful to assist patients in obtaining safer CAR-T cell therapy. NK cell therapy is also an important strategy of immune cell therapy, but there are many bottlenecks and limitations. Zhang et al. tried to explore the synergistic NK cell therapy, and the experimental results proved that fucosymulation helped promote NK cell infiltration in the B-cell tumor microenvironment and improve the function of NK cells. It has important reference value for the future exploration of NK cell therapy. The treatment of peripheral T cell lymphoma is facing challenges. HDAC has achieved good efficacy in the treatment of refractory and relapsed PTCL. Wang et al. (Front Immunol. 2022 Feb 2;13:835103.) explored the feasibility of first-line treatment of PTCL with chidamide combined with chemotherapy. The results show that the combination of chidamide can improve the progression-free survival of patients with peripheral T cell lymphoma, and the safety is controllable.

On the whole, this special issue brings together high-quality manuscripts, exploring some unresolved clinical problems of LPD, with value for accurate diagnosis, prognosis, and innovative treatments in the future.

## Author contributions

The author confirms being the sole contributor to this work and has approved it for publication.

## Acknowledgments

We sincerely thank all the contributing authors to this collection.

## Conflict of interest

The author declares that the research was conducted in the absence of any commercial or financial relationships that could be construed as a potential conflict of interest.

## Publisher’s note

All claims expressed in this article are solely those of the authors and do not necessarily represent those of their affiliated organizations, or those of the publisher, the editors and the reviewers. Any product that may be evaluated in this article, or claim that may be made by its manufacturer, is not guaranteed or endorsed by the publisher.

